# Inteligência epidemiológica, investimento em tecnologias da informação e as novas perspectivas para o uso de dados na vigilância em saúde

**DOI:** 10.1590/0102-311XPT160523

**Published:** 2024-09-09

**Authors:** Débora Medeiros de Oliveira e Cruz, Caroline Dias Ferreira, Luciana Freire de Carvalho, Valéria Saraceni, Betina Durovni, Oswaldo Gonçalves Cruz, Marcio Henrique de Oliveira Garcia, Gislani Mateus Oliveira Aguilar

**Affiliations:** 1 Secretaria Municipal de Saúde do Rio de Janeiro, Rio de Janeiro, Brasil.; 2 Instituto de Educação Médica, Rio de Janeiro, Brasil.; 3 Universidade Federal do Rio de Janeiro, Rio de Janeiro, Brasil.; 4 Programa de Computação Científica, Fundação Oswaldo Cruz, Rio de Janeiro, Brasil.; 5 Ministério da Saúde, Brasília, Brasil.

**Keywords:** Vigilância em Saúde Pública, Monitoramento Epidemiológico, Epidemias, Public Health Surveillance, Epidemiological Monitoring, Epidemics, Vigilancia en Salud Pública, Monitoreo Epidemiológico, Epidemias

## Abstract

No Município do Rio de Janeiro, Brasil, a incorporação do conceito de inteligência epidêmica e de recursos tecnológicos sustentou novas perspectivas para a utilização de dados pela vigilância em saúde, a partir da pandemia de COVID-19. Neste artigo apresenta-se o Centro de Inteligência Epidemiológica (CIE), ferramentas e produtos desenvolvidos na coordenação. O CIE foi inaugurado em março de 2022, com equipe multiprofissional, apoiado nas premissas de transparência e integração de diversas fontes de dados para detecção precoce de mudanças nas tendências de eventos de importância em Saúde Pública. A aquisição inicial de um *data lake* favoreceu mudanças nos processos de consumo, gerenciamento e segurança para os dados processados. Esse *data lake* armazena, atualmente, a Base Carioca - uma coorte retrospectiva composta de indivíduos com histórico vacinal para COVID-19 e/ou eventos relacionados à doença. Painéis descritivos e analíticos foram desenvolvidos e disponibilizados, respectivamente, para uso público e para os gestores da vigilância em saúde. Um painel de alertas, voltado ao monitoramento de tendências nos atendimentos da rede de urgência e emergência municipal, foi implantado e subsidiou ações de resposta rápida nos territórios da cidade. O CIE desenvolveu o conceito de inteligência epidemiológica no Sistema Único de Saúde, e essa mudança de paradigma tornou-se possível em função de investimentos em recursos físicos/humanos, integração de métodos epidemiológicos, estatísticos e das ciências de dados, além de incorporação de fontes de dados diferenciadas nas análises de dados.

## Abstract (en)

In the municipality of Rio de Janeiro, Brazil, the incorporation of the concept of epidemic intelligence and technological resources has supported new perspectives for the use of data by health surveillance, since the COVID-19 pandemic. This article presents the Epidemiological Intelligence Center (CIE) and the tools and products developed in its coordination. The CIE was inaugurated in March 2022, with a multiprofessional team, supported by the premises of transparency and integration of various data sources for early detection of changes in the trends of events of importance in Public Health. The initial acquisition of a data lake favored changes in the consumption, management and security processes for the data processed. This data lake currently stores the Carioca Base - a retrospective cohort of individuals with a history of COVID-19 vaccination and/or events related to the disease. Descriptive and analytical dashboards have been developed and made available, respectively for public use and for health surveillance administrators. An alert panel, aimed at monitoring trends in care in the urgency and emergency network, was implemented and subsidizes rapid response actions in the city’s territories. The CIE developed the concept of epidemiological intelligence in the Brazilian Unified National Health System and this paradigm shift was made possible by investments in physical/human resources, the integration of epidemiological, statistical and data science methods, as well as the incorporation of different data sources in data analysis.

## Abstract (es)

En el municipio de Río de Janeiro, Brasil, la inclusión del concepto de inteligencia epidémica y de recursos tecnológicos favoreció nuevas perspectivas en el uso de datos por parte de la vigilancia sanitaria desde la pandemia del COVID-19. Este artículo presenta el Centro de Inteligencia Epidemiológica (CIE), las herramientas y los productos desarrollados en coordinación. El CIE se creó en marzo de 2022 con un equipo multidisciplinar bajo las premisas de transparencia e integración de diversas fuentes de datos para la detección temprana de cambios en las tendencias a grandes eventos en Salud Pública. La adquisición inicial de un *data lake* promovió cambios en los procesos de consumo, gestión y seguridad de los datos procesados. Este *data lake* almacena actualmente la Base Carioca, una cohorte retrospectiva compuesta por individuos con antecedentes de vacunación contra el COVID-19 y/o eventos relacionados con la enfermedad. Se desarrollaron paneles descriptivos y analíticos, y se los pusieron a disposición, respectivamente, para uso público y para los gerentes de la vigilancia sanitaria. Se implementó un panel de alerta, dirigido a monitorear las tendencias a la asistencia en la red de urgencia y emergencia del municipio, el cual subvenciona acciones de pronta respuesta en los territorios de la ciudad. El CIE desarrolló el concepto de inteligencia epidemiológica en el Sistema Único de Salud, y este cambio de paradigma se hizo posible gracias a las inversiones en recursos físicos/humanos, la integración de métodos epidemiológicos, estadísticos y de ciencia de datos, además de la inclusión de fuentes de datos diferenciadas en el análisis de datos.

## Introdução

A pandemia de COVID-19 intensificou a necessidade de elaboração de informações oportunas para o processo decisório no setor saúde. No Município do Rio de Janeiro, Brasil, foi ativado, no início de 2021, o Centro de Operações de Emergências (COE) COVID-19 Rio para coordenar as estratégias de resposta a essa emergência de saúde pública ^1^ ^,^ ^2^.

As informações relacionadas à COVID-19 produzidas nesse centro foram inovadoras ao incluir análises dos registros de vacinados oriundos do Sistema de Informação do Programa Nacional de Imunizações (SI-PNI) e dados de atendimentos da rede de urgência e emergência municipal ^3^. Apesar dos esforços realizados para o monitoramento do agravo naquele momento, as abordagens tradicionais da vigilância epidemiológica permanecem como um desafio, particularmente em eventos emergentes e reemergentes ^4^.

O contínuo desenvolvimento das tecnologias de informação e a oferta de diferentes ferramentas e métodos para o armazenamento, processamento e análise de dados (estruturados e não estruturados) contribui para a ciência de dados populacionais e pode impactar as práticas de vigilância em saúde ^5^. Especialmente na pandemia de COVID-19, lacunas relacionadas à infraestrutura de tecnologia constituíram-se em entraves às atividades de preparação e resposta às emergências de saúde pública ^6^.

A experiência adquirida no COE COVID-19 Rio sinalizou uma política de governança favorável à concepção de uma nova unidade de trabalho, orientada ao desenvolvimento e incorporação de técnicas e ferramentas mais modernas para gestão, no âmbito da vigilância em saúde. Pelo exposto, este artigo pretende apresentar o CIE, ferramentas e produtos desenvolvidos pela coordenação, a partir de sua implantação.

## O Centro de Inteligência Epidemiológica

O CIE foi implantado no Município do Rio de Janeiro em março de 2022 e subordinado ao organograma da Superintendência de Vigilância em Saúde. A equipe multiprofissional dessa coordenação é composta por epidemiologistas, cientistas de dados, estatísticos e programadores e um geógrafo. Esses profissionais têm experiência na área de vigilância em saúde e compartilham o uso de linguagens computacionais para a execução dos processos de trabalho, principalmente a linguagem R.

Sua estruturação teve como premissas a necessidade de transparência e eficiência na utilização dos dados públicos, o investimento em infraestrutura tecnológica e a operacionalização de inteligência para epidemias. Esse conceito de inteligência para epidemias, do inglês *epidemic intelligence*, inicialmente priorizou o desenvolvimento de ferramentas e a inovação em análises de dados voltadas à detecção precoce de eventos de importância em Saúde Pública e monitoramento de tendências de eventos com potencial para tornar-se emergência de saúde pública, buscando antecipar a atuação da vigilância em saúde ^7^ ^,^ ^8^.

O investimento em tecnologias - um dos pilares de trabalho na coordenação - foi fundamental à integração e aprimoramento do uso das bases de dados. Nesse sentido, um projeto para a aquisição e estruturação de um *data lake* foi uma das primeiras estratégias adotadas pelo CIE.

## O *data lake*

Para melhorar a eficiência no processamento de registros, flexibilizar e diversificar a possibilidade de cruzamento de dados, um *data lake* foi implantado na coordenação. A importância desse ambiente para a vigilância em saúde derivou da necessidade de armazenamento de registros de fontes e estruturas diversas e da facilitação de uma política de gerenciamento mais segura e uniforme para os dados ^9^.

No *data lake* do CIE, as tabelas oriundas dos sistemas de informação em saúde (SIS) tradicionais e outras fontes de dados municipais são populadas seguindo etapas de extração, transformação e carregamento de dados. Para a etapa de extração, estratégias incrementais (para captura de dados via sistema API do SI-PNI; rede de urgência e emergência municipal; Sistema de Informação de Agravos de Notificação (SINAN), dados vitais, dados da atenção primária) e completas (dados relacionados às arboviroses; síndrome respiratória aguda grave - SRAG, entre outros) têm sido aplicadas. As rotinas de transformação dos dados incluem: (i) limpeza; (ii) enriquecimento de dados; (iii) normalização; e (iv) agregação. Em relação ao carregamento, o *data lake* recebe conjuntos de dados parciais e, em alguns casos, a base completa é substituída (para manutenção da integridade dos dados).

**Figura 1:**
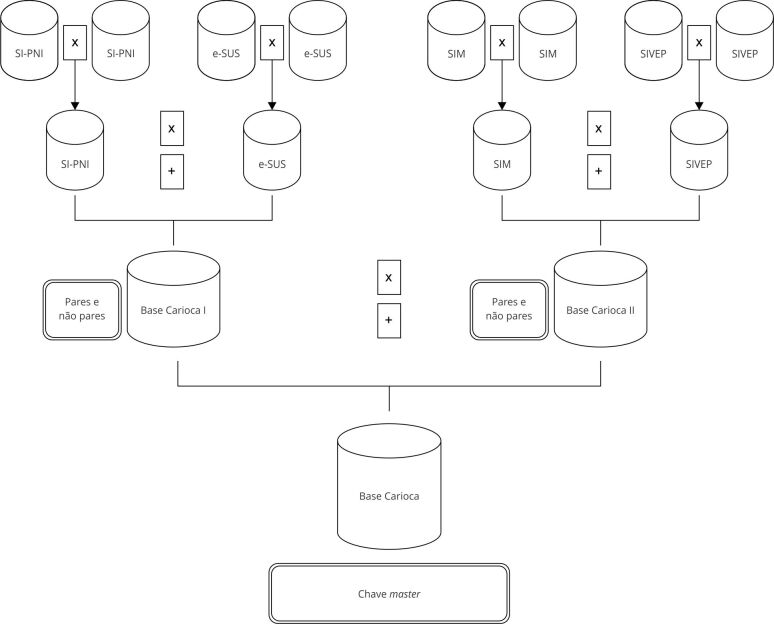
Bases de dados utilizadas para a obtenção da Base Carioca.

SIM: Sistema de Informações de Mortalidade; SI-PNI: Sistema de Informação do Programa Nacional de Imunizações; SIVEP: Sistema de Vigilância Epidemiológica; SUS: Sistema Único de Saúde.

Dados do Sistema de Informações sobre Mortalidade (SIM), SIVEP-Gripe (Sistema de Vigilância Epidemiológica da Gripe), e-SUS e SI-PNI foram vinculados e estão armazenados no *data lake*. O relacionamento de bases de dados é uma metodologia bastante utilizada para a pesquisa, e sua aplicação no campo da vigilância em saúde tem aumentado ao longo dos anos ^10^ ^,^ ^11^ ^,^ ^12^. Técnicas de *linkagem* de dados foram empregadas para a obtenção da Base Carioca, uma coorte digital (retrospectiva) com cerca de 7 milhões de registros de indivíduos vacinados e/ou que tiveram eventos relacionados à COVID-19 no Município do Rio de Janeiro. Os casos de síndrome gripal, SRAG, óbitos e vacinados foram vinculados por algoritmo hierárquico determinístico, conforme previamente indicado por Pacheco et al. ^13^. Nesse processo foi obtida uma chave *master*, cujo código final permitiu acompanhar o histórico do indivíduo, a partir da imunização, adoecimento e/ou morte. Na Figura 1, estão resumidas as bases utilizadas no processo de vinculação até a obtenção da coorte.

## O Epi Rio e a plataforma interna de painéis epidemiológicos

Alinhado ao pressuposto da transparência pública das informações em saúde do município, o primeiro produto desenvolvido no CIE foi um observatório epidemiológico (Epi Rio) disponível na Internet, com painéis descritivos e interativos. Nesse espaço digital, semanalmente, os dados relacionados a diferentes agravos/doenças, assim como eventos vitais do Município do Rio de Janeiro, são atualizados e disponibilizados no endereço eletrônico: https://epirio.svs.rio.br/. Os painéis do Epi Rio têm sido consultados para atividades de ensino e pesquisa, gestão pública e fornecem também subsídios para o encaminhamento de respostas à mídia, facilitando as atividades de comunicação em saúde.

Além dos painéis descritivos, a coordenação incorporou às rotinas de análise da vigilância em saúde modelos estatísticos voltados à correção de atrasos de notificação (*nowcasting*) e previsão de tendências para eventos em saúde. Ganhos tangíveis derivados da utilização de modelagem hierárquica bayesiana para correção de atrasos na notificação de casos foram apresentados previamente por Bastos et al. ^14^, sendo essa metodologia incorporada pela coordenação para a correção de atrasos nas notificações de arboviroses e COVID-19.

No CIE, métodos de modelagem preditiva foram direcionados a diversos eventos/agravos, tais como síndrome gripal, febre, cefaleia, mialgia, dengue, SRAG. As séries temporais foram elaboradas a partir de registros oriundos da rede de urgência e emergência municipal e também SIS tradicionais, como, por exemplo, o SINAN.

Para essa modelagem tem sido utilizados modelos ARIMA e XGboost, com painéis para visualização construídos sob a linguagem R e o pacote Shiny. Os modelos são aplicados às séries temporais e comparados conforme ajuste e métricas de performance. Os painéis com os modelos preditivos e nowcasting foram disponibilizados em plataforma interna para os técnicos da vigilância em saúde e gestores da Secretaria Municipal de Saúde do Rio de Janeiro.

## O Painel de Alertas

A utilização dos registros de atendimento da rede de urgência e emergência do município foi iniciada na pandemia de COVID-19, diante da observação de que as informações oriundas dessa fonte antecipavam a tendência de elevação do número de casos em relação aos dados dos SIS tradicionais. Esse acompanhamento configurou-se em uma atividade inovadora dentro do processo de trabalho da vigilância em saúde e foi ampliado para outros eventos em saúde ^3^.

O monitoramento dos registros da rede de urgência e emergência municipal tem sido realizado no CIE para explorar padrões de ocorrência de sinais/sintomas em atendimentos realizados nas unidades de urgência/emergência no município. O processo de trabalho para a elaboração dessa informação envolve rotinas de processamento e modelagem estatística dos registros para a identificação precoce de alterações em 12 séries monitoradas.

Modelos aditivos generalizados (GAM) foram utilizados para remoção do ruído presente nas séries (agregação diária) e extração de uma tendência geral. Foram aplicadas métricas para indicar a tendência de crescimento ou queda apoiadas nos cálculos dos quantis históricos dos casos. As séries que estão acima de patamares preestabelecidos são sinalizadas para as equipes da vigilância, determinando ações de resposta rápida no território.

**Figura 2:**
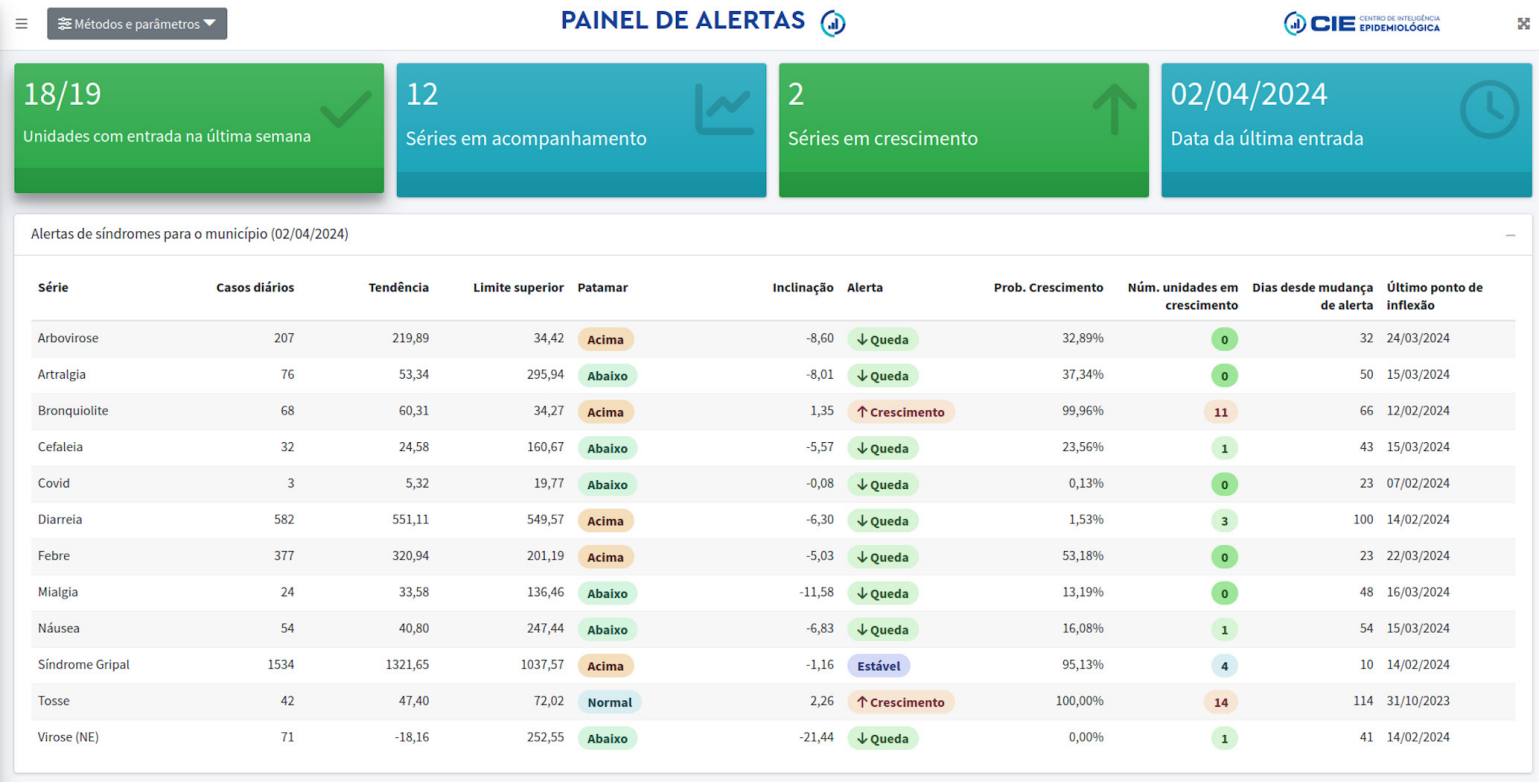
Tela principal do Painel de Alertas, abril de 2024, Município do Rio de Janeiro, Brasil.

O desenvolvimento dessa estratégia favorece a detecção precoce de surtos, permite a divulgação de alertas para a rede de saúde e orienta respostas mais oportunas a possíveis situações de emergência. A consolidação das tendências da rede de urgência e emergência no município foi estruturada no formato de um painel e disponibilizada em plataforma interna (Figura 2). A informação sobre a situação de séries em crescimento (alertas epidemiológicos) é organizada e reiterada pelos epidemiologistas do CIE no formato de boletins denominados *Sala Zero*, relatório encaminhado aos gestores da vigilância em saúde.

## A vigilância de arboviroses e as estratégias automatizadas de monitoramento

O acompanhamento da situação das arboviroses é uma atividade prioritária para a vigilância em saúde, diante da magnitude dos casos, circulação de dengue e chikungunya e o risco de reaparecimento da Zika no Município do Rio de Janeiro ^15^ ^,^ ^16^. Para melhorar a eficiência das análises relacionadas às arboviroses, foram desenvolvidos no CIE relatórios automatizados e ferramentas digitais para os dados epidemiológicos e ambientais da vigilância em saúde.

Dados provenientes do monitoramento entomológico e do *Levantamento de Índice Rápido para o Aedes aegypti* (LIRAa) foram disponibilizados em um boletim entomológico no formato HTML. Esses dados são consolidados pela Coordenação de Vigilância Ambiental em Saúde e enviados ao CIE mensalmente para normalização e automatização das análises descritivas.

Relatórios relacionados aos casos de arboviroses notificados ao SINAN foram automatizados, e a informação é atualizada e disponibilizada semanalmente pela coordenação no endereço eletrônico: https://epirio.svs.rio.br/painel/arboviroses/.

**Figura 3:**
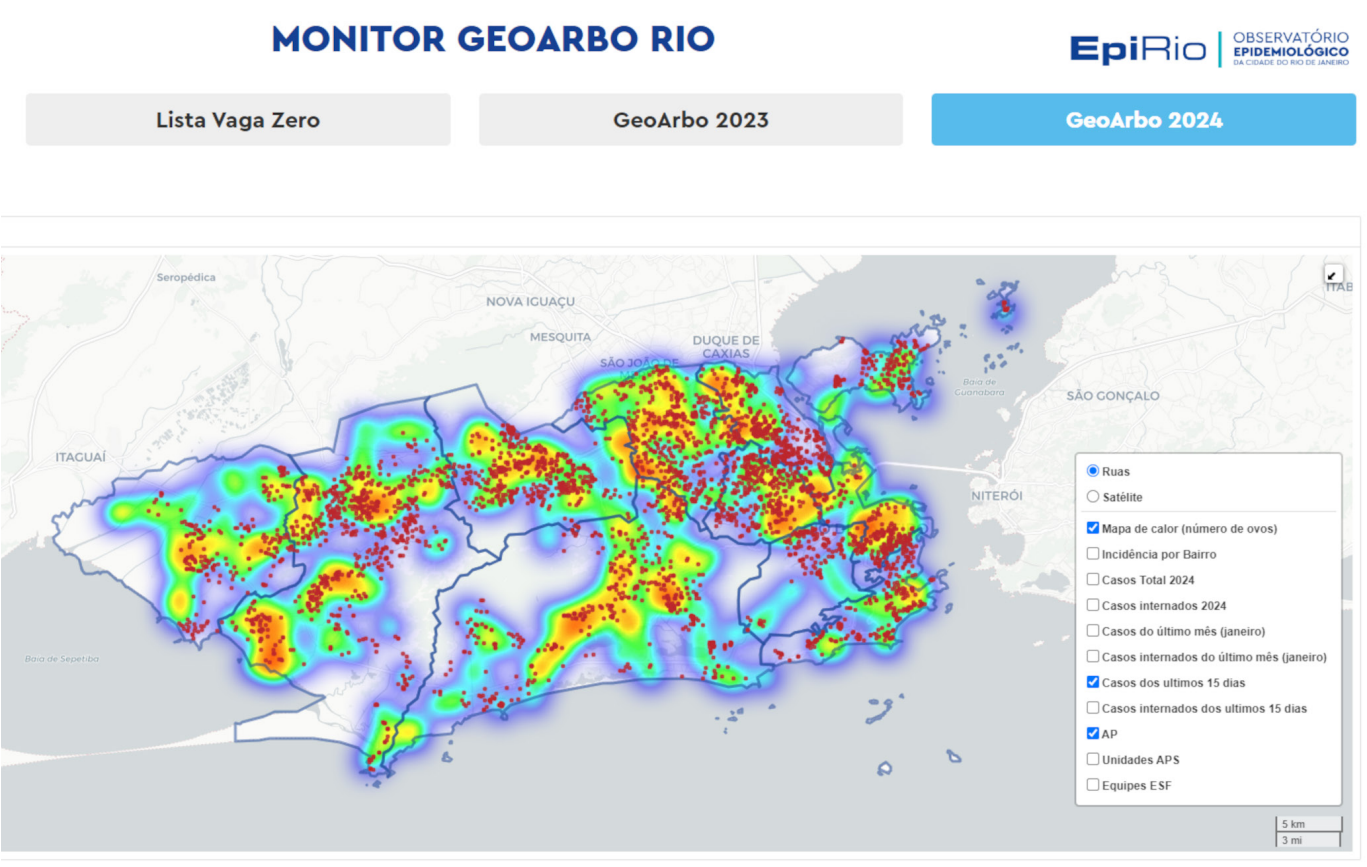
Tela principal do GEOARBO, março de 2024, Município do Rio de Janeiro, Brasil.

Por fim, com o objetivo de facilitar a visualização das informações relacionadas às arboviroses, foi desenvolvido o GEOARBO, um mapa interativo para monitoramento dos dados epidemiológicos e entomológicos (Figura 3). Essa ferramenta tem sido amplamente utilizada nas reuniões da sala de situação, nas quais são discutidas as estratégias de enfrentamento às arboviroses. O GEOARBO fornece a localização geográfica dos casos e está disponível a todos os profissionais da rede de saúde do município para consulta.

## Considerações finais

As iniciativas apresentadas anteriormente promoveram uma mudança de paradigma em processos essenciais da vigilância em saúde: consumo e processamento de dados, análise e divulgação de informações. A utilização de registros de prontuários eletrônicos (rede de urgência e emergência municipal) tem se mostrado importante para as atividades de resposta da vigilância, somando às abordagens tradicionais (vigilância passiva). Futuramente, idealiza-se que as análises de dados possam incorporar mais fontes, como, por exemplo, mídias sociais e imagens e, também, técnicas orientadas para detecção de padrões em textos.

A partir do *data lake*, foi possível melhorar o processamento e a segurança no armazenamento de registros. A vinculação de dados estruturados na Base Carioca abre oportunidades para a elaboração de projetos para estudos longitudinais (por meio de uma coorte digital), implica em novas possibilidades de análise e uso para os sistemas de registro administrativo do setor saúde, e amplia perspectivas para as investigações da infecção por COVID-19 como um fator de risco para outras doenças.

A estruturação do Epi Rio facilitou as atividades de interação com os dados públicos dos SIS por diferentes usuários e atende a necessidade de publicização das informações em saúde. A aplicação de técnicas de *nowcasting* às notificações e modelagem preditiva foi ampliada a outros eventos de importância em saúde, como as arboviroses - cujas informações estão consolidadas em relatórios automatizados e no GEOARBO. O desenvolvimento do Painel de Alertas permitiu a identificação de possíveis emergências de saúde pública, geração de alertas precoces e respostas mais oportunas a esses eventos.

O CIE é uma iniciativa pioneira no país. Sua atuação tem oferecido suporte para a gestão da rede de vigilância em saúde e impacta positivamente a saúde coletiva ao incorporar inovações digitais ao Sistema Único de Saúde (SUS). Além disso, dialoga com a Política Nacional de Informação e Informática em Saúde, ao materializar, por meio de suas ferramentas e produtos, informação destinada ao cidadão, trabalhadores e gestores da saúde.

Destaca-se, nesse ponto, a importância de indicar a ampliação do conceito de inteligência para epidemias materializado no CIE, pois o trabalho realizado na coordenação não se limitou à identificação de surtos e emergência de saúde pública, antes buscou adequar-se às necessidades dos territórios e da vigilância em saúde do Município do Rio de Janeiro, operacionalizando inteligência epidemiológica para o monitoramento de situações que afetam a população numa perspectiva ampliada.

Por fim, entende-se que o desenvolvimento do trabalho do CIE tem potencial para gerar informações inovadoras para o SUS, intensifica o movimento de ciência de dados populacionais, fortalece a vigilância em saúde e reforça o processo de decisões orientado por informações mais oportunas.
